# Validation of abridged mini-mental state examination scales using population-based data from Sweden and USA

**DOI:** 10.1007/s10433-016-0394-z

**Published:** 2016-08-20

**Authors:** Malin Christina Ericsson, Margaret Gatz, Ingemar Kåreholt, Marti G. Parker, Stefan Fors

**Affiliations:** 1 Department of Medical Epidemiology and Biostatistics, Karolinska Institutet, Stockholm, Sweden; 2 Department of Psychology, University of Southern California, Los Angeles, USA; 3 Aging Research Center, Karolinska Institutet & Stockholm University, Stockholm, Sweden; 4 Institute for Gerontology, School of Health and Welfare, Jönköping University, Jönköping, Sweden

**Keywords:** Oldest old, Surveys, Cognition, MMSE

## Abstract

The objective of this study is to validate two abridged versions of the mini-mental state examination (MMSE): one intended for use in face-to-face interviews, and the other developed for telephonic interviews, using data from Sweden and the US to validate the abridged scales against dementia diagnoses as well as to compare their performance to that of the full MMSE scale. The abridged versions were based on eight domains from the original MMSE scale. The domains included in the MMSE-SF were registration, orientation, delayed recall, attention, and visual spatial ability. In the MMSE-SF-C, the visual spatial ability item was excluded, and instead, one additional orientation item was added. There were 794 participants from the Swedish HARMONY study [mean age 81.8 (4.8); the proportion of cognitively impaired was 51 %] and 576 participants from the US ADAMS study [mean age 83.2 (5.7); the proportion of cognitively impaired was 65 %] where it was possible to compare abridged MMSE scales to dementia diagnoses and to the full MMSE scale. We estimated the sensitivity and specificity levels of the abridged tests, using clinical diagnoses as reference. Analyses with both the HARMONY and the ADAMS data indicated comparable levels of sensitivity and specificity in detecting cognitive impairment for the two abridged scales relative to the full MMSE. Receiver operating characteristic curves indicated that the two abridged scales corresponded well to those of the full MMSE. The two abridged tests have adequate validity and correspond well with the full MMSE. The abridged versions could therefore be alternatives to consider in larger population studies where interview length is restricted, and the respondent burden is high.

**Electronic supplementary material:**

The online version of this article (doi:10.1007/s10433-016-0394-z) contains supplementary material, which is available to authorized users.

## Introduction

The mini-mental state examination (MMSE) is a test widely used to screen for cognitive impairment as well as to track development of cognitive function over time (Molloy and Standish 1997). The test comprises tasks that examine various cognitive functions and is relatively easily administered (Folstein et al. 1975).

Panel studies and other large data collections among older adults are dependent on efficient design to ensure high response rate and participation in follow-ups. Restrictions in time and magnitude may be crucial as extensive interviews can be tiring for the participant (Lundberg and Thorslund 1996). Different methods of collecting data might therefore be required in order to obtain a representative sample (Kelfve et al. 2013). Such methods may entail face-to-face interviews, interviews by telephone, and by proxy interviews. Alternatives to direct interviews may enable participation by individuals that otherwise may be constrained due to age or impairments (Fong et al. 2009).

Different cognitive screening tests are commonly used in studies of older adults, and the MMSE is one of the most well-known and used tests. However, with the exception of MMSE and a few others, they are rarely validated (Cullen 2007). Several short forms and versions of MMSE have been found to correspond well to the original MMSE which, even if concise, may sometimes be too extensive to include in multipurpose surveys. The accuracy of previous short versions has depended on the items included, where the cutoff was set and, to some extent, how and in what context the version was used (Davies and Larner 2013).

The two abridged versions tested in this study were developed for use in multipurpose studies. They were initially designed for the SWEOLD study: a Swedish panel study with a representative sample from the older population (≥77 years) (Lennartsson et al. 2014). Abridged versions were needed because SWEOLD data collection was broad in scope with very limited time allocated to cognitive screening. Moreover, the advanced age of the population created a concern that many might be exhausted by lengthy interviews. Items were selected from the standard, while Swedish full scale (Palmqvist et al. 2013). The selection of items drew in part on an earlier study by Braekhus et al. (1992) to identify the most efficient items for identifying cognitive impairment. Selection was further guided by theoretical (e.g. to include most of the cognitive domains) and pragmatic considerations (e.g., ease of administration, time constraints) (Parker et al. 1996). These abridged versions have been used in a range of research papers, using the average score (Fors et al. 2009; Parker et al. 2013), a cutoff (Meinow et al. 2011), or both (Andel et al. 2007, 2011).

The aim of this paper is to present a validation of the two abridged MMSE scales, using data from two large nationally representative studies that include cognitive screening data and clinical diagnosis of dementia: the study of dementia in Swedish twins (HARMONY) in Sweden, and the aging, demographics, and memory study (ADAMS), in the US. One of the abridged scales is intended for use in face-to-face interviews (MMSE-SF), and the other is a complementary version which does not require any physical engagement from the respondent, making it viable for use in telephonic interviews (MMSE-SF-C).

## Methods

The data used for the analyses are from two different national panel studies: HARMONY (Gatz et al. 2005) and ADAMS (Langa et al. 2005). Both are substudies, focused on cognitive impairment and dementia, with population samples derived from two large national cohort studies: the Swedish Twin Registry (Lichtenstein et al. 2002) and the Health and Retirement Study (Sonnega et al. 2014).

Participation in HARMONY included an initial screening phase and a subsequent clinical phase. The clinical phase consisted of both in-home physical examination and neuropsychological testing. A final clinical diagnosis was given in accordance with DSM-IV. The diagnosis allowed for three outcome categories: dementia, questionable dementia and no dementia (Gatz et al. 2005). Questionable dementia corresponds to meeting two of the three DSM-IV diagnostic criteria for dementia (impaired memory, other cognitive disturbance, and difficulties in functioning). In the present study, we included participants of 75 years or older who completed the clinical phase (*n* = 794).

The clinical assessment in ADAMS contains a large variety of neuropsychological tests. The clinical diagnosis was based on DSM-III-R and DSM IV. There were three outcome categories: dementia, cognitive impairment with no dementia (CIND), and no dementia. CIND was defined as self or informant reported cognitive impairment that did not meet the criteria for dementia or reached the threshold for impairment in each cognitive domain (Langa et al. 2005). In this study, we included only those who were 75 years or older (*n* = 648). On account of a variation in completion of the different items, the sample size also differed slightly between the full MMSE (*n* = 576), the MMSE-SF (*n* = 594) and the MMSE-SF-C (*n* = 638). However, only subjects with no missing data from the full MMSE test were included in the analyses.

The full MMSE covers 11 domains: registration, orientation, recall, attention, or calculation (serial sevens or spelling), naming, repetition, comprehension (verbal and written), writing, and construction. The items included in the abridged versions are shown in Table 1. The items that were applied in the two abridged versions were compiled from the full versions that were available in both HARMONY and ADAMS. The scoring for registration and attention was adjusted to be in better proportion to the other items. For registration, a correct repetition of all three words was scored as one point. For attention (serial sevens), each correct answer was given 0.4 points for a maximum of two points. The two abridged versions are identical except for one item. The MMSE-SF included the task of copying a figure. The MMSE-SF-C instead included one additional orientation item.

**Table 1:** Overview of items included in the two abridged versions of MMSE (max score 11 in both abridged versions)

MMSE-SF		MMSE-SF-C	
Registration		Registration	
Repeat objects	1	Repeat objects	1
Orientation		Orientation	
Year	1	Weekday	1
Month	1	Year	1
Date	1	Month	1
Country/State	1	Date	1
Delayed recall		Country/State	1
Repeat objects	3	Delayed recall	
Attention		Repeat objects	3
Serial sevens	2	Attention	
Visual spatial ability		Serial sevens	2
Draw a figure	$1$		

The abridged scoring algorithms were applied to corresponding MMSE items from both HARMONY and ADAMS, such that each subject had scores on the full MMSE and the two abridged versions. The orientation item State was not included in the HARMONY data collection and was instead replaced with the item Country. These two items had comparable percentage of correct responses (in HARMONY, Country 91 % and in ADAMS, State 90 %).

The MMSE was treated both as a continuous and a dichotomous variable. In the dichotomized alternative, a cutoff was applied to separate the cognitively impaired and the cognitively nonimpaired, while a <24 cutoff for dementia is commonly used (Bassett and Folstein 1991), as there is no conclusively defined cutoff for the MMSE. Analyses were performed to estimate optimal cutoffs based on the best-compiled outcome from a range of sensitivity and specificity levels when testing the continuous scale against a dichotomous test of reference. Optimal cutoffs were performed using the *roctg* command in STATA. Initial analyses used the clinical diagnosis as the reference test in each dataset. Subsequently, analyses were performed using the full MMSE as reference to the abridged scales. Based on the aggregated results from these analyses, cutoffs of <24 for the full scale and <8 for the two abridged scales were adopted for evaluating sensitivity and specificity.

The clinical diagnosis in each dataset was used as the reference test in the analyses for validity. The three subcategories; normal cognitive function, questionable dementia/CIND, and dementia were recoded into a dichotomous variable where dementia and questionable dementia/CIND both were coded as presence of disease.

### Statistical analyses

Difference in proportions of cognitively impaired in the abridged scales compared with the full MMSE were assessed with Chi-square tests and Fisher´s exact test. In order to test the validity of the two abridged scales, sensitivity and specificity levels as well as the receiver operating characteristics (ROC) were calculated. The analysis of sensitivity and specificity shows the agreement between the applied cutoffs and a clinical diagnosis. Sensitivity is the rate of subjects with the condition that also get a positive test result. Specificity is the rate of subjects without the condition that get a negative test result. Based on this, the positive predictive value (PPV) and the negative predictive value (NPV) can be estimated. PPV gives the probability that a positive test also means that there is the presence of disease. NPV, on the other hand, is the probability that a negative test means that there is the absence of disease.

The ROC curve graphically shows the validity conditions by testing the whole scale and its agreement with other alternative tests as well as the reference test. ROC analysis was carried out in order to test how the abridged MMSE scales corresponded to the full MMSE scale in terms of accuracy with the reference test. The three tests were tested simultaneously against the clinical diagnosis but separately for the two datasets. A numerical value can be calculated to describe the area under the ROC curve (AUC). The AUC is a value derived from the unit square but as 0.5 equals a random result, the AUC value will be between 0.5 and 1.0, while 1.0 is equivalent to full correspondence to the test of reference (Fawsett 2006).

## Results

Demographic characteristics of the HARMONY and ADAMS samples, and mean scores and proportions scoring below cutoffs on the full MMSE and the two abridged scales, for the total sample and for demographic and diagnostic subsets, are presented in Table 2. There were statistically significant differences between the proportions of the populations classified as cognitively impaired with any of the short forms compared with the full MMSE, with the exception of the dementia category in the ADAMS sample regarding the MMSE-SF-C. In general, the full MMSE classified slightly more participants as cognitively impaired compared with the abridged forms.

**Table 2:** Statistics of HARMONY (*n* = 794) and ADAMS (*n* = 576), including the cognitive scales, the full MMSE (0–30), the MMSE-SF (0–11), and MMSE-SF-C (0–11)

	Total		Mean score on abridged MMSE				Proportion of impaired based on MMSE cutoffs (%)					
	HARMONY	ADAMS	HARMONY		ADAMS		HARMONY			ADAMS		
	*n* (%)	*n* (%)	MMSE-SF mean (SD)	MMSE-SF-C mean (SD)	MMSE-SF mean (SD)	MMSE-SF-C mean (SD)	Full MMSE (<24)	MMSE-SF (<8)	MMSE-SF-C (<8)	Full MMSE (<24)	MMSE-SF (<8)	MMSE-SF-C (<8)
Gender												
Female	472 (59.5)	343 (59.5)	5.8 (3.4)	5.9 (3.5)	6.4 (3.2)	6.6 (3.3)	62.1	56.7*	54.2*	60.9	58.9*	53.4*
Male	322 (40.5)	233 (40.5)	6.7 (3.1)	6.7 (3.1)	7.1 (2.8)	7.3 (2.7)	49.1	49.4*	46.6*	53.2	55.4*	49.8*
Age (years)												
75–79	308 (38.8)	171 (29.7)	6.9 (3.1)	6.9 (3.1)	7.6 (2.7)	7.9 (2.6)	48.1	45.1*	41.9*	46.2	45.6*	37.4*
80–84	269 (33.9)	193 (33.5)	6.3 (3.3)	6.3 (3.4)	7.0 (3.1)	7.3 (3.1)	56.1	51.7*	49.1*	52.3	52.3*	45.6*
85–89	168 (21.1)	116 (20.1)	5.1 (3.3)	5.1 (3.4)	6.0 (3.0)	6.3 (3.1)	68.4	66.1*	64.3*	66.4	66.4*	63.8*
≥90	49 (6.2)	96 (16.7)	5.2 (3.2)	5.3 (3.3)	5.0 (3.1)	5.3 (3.1)	75.5	75.6*	75.5*	79.2	78.1*	76.0*
Education (years)												
≤8	555 (71.3)	201 (34.9)	5.8 (3.3)	5.9 (3.4)	5.2 (2.6)	5.7 (2.7)	61.3	56.6*	54.8*	85.1	82.6*	72.6*
9–12	181 (23.3)	222 (38.5)	7.2 (3.0)	7.3 (3.1)	7.0 (3.1)	7.2 (3.1)	42.5	42.5*	38.1*	50.9	51.4*	46.4*
≥13	42 (5.4)	153 (26.6)	6.9 (3.2)	6.8 (6.8)	8.1 (3.0)	8.1 (3.1)	45.2	50.0*	50.0*	32.0	33.3*	32.7*
Clinical diagnosis												
Normal cognitive function	390 (49.1)	204 (35.4)	8.7 (1.5)	8.8 (1.5)	9.3 (1.5)	9.5 (1.3)	33.1	28.0*	25.9*	18.1	17.2*	12.3*
Questionable dementia/CIND^a^	111 (14.0)	178 (30.9)	6.3 (1.9)	6.4 (2.1)	7.2 (2.0)	7.6 (1.9)	82.9	78.4*	73.0*	61.2	59.6*	48.9*
Dementia	293 (36.9)	194 (33.7)	2.4 (2.4)	2.8 (2.4)	3.5 (2.1)	3.6 (2.2)	98.3	98.0*	97.6*	96.4	97.9*	96.4

* The proportions were statistically significantly different from the full MMSE (*p* < 0.05)

^a^Cognitive impairment with no dementia

As the two datasets had different criteria for including participants in the in-home assessment for dementia, and different diagnostic criteria for the middle category, the proportions with dementia and questionable dementia/CIND differed. In the HARMONY data, 49.1 % were diagnosed as not having dementia, while 14 % were questionable, and 36.9 % had dementia. In the ADAMS data, 35.4 % were diagnosed as not having dementia, 30.9 % as CIND, and 33.7 % as having dementia.

Mean values of MMSE scores and the proportion of cognitive impairment, based on the appointed cutoffs (<24 and <8), varied in relation to demographic factors. The MMSE cutoffs corresponded predictably to the categories of clinical diagnosis. As seen in the right-hand half of Table 2, among those with dementia, more than 95 % scored below the MMSE cutoff. Among those with normal cognitive function, about 25–33 % in the HARMONY sample and 12–18 % in the ADAMS sample scored below the MMSE cutoff.

The rates of sensitivity and specificity are presented in Table 3, comparing MMSE cutoffs to clinical diagnosis. In both datasets, the measured values of sensitivity, specificity, PPV, and NPV were similar for the full MMSE and for both abridged versions. In the analyses with the HARMONY data, sensitivity levels were overall high (>90 %), while the levels of specificity were lower. Levels of PPVs and NPVs were also consistent across tests. Sensitivity levels from the ADAMS analyses were moderate (<80 %) in all the three versions of the test, while specificity rates were higher. The PPVs in the ADAMS data were comparably high, while the NPVs were lower in comparison (<70 %); however, the levels did not differ much between versions. Significance testing of similarity between the tests within each dataset showed that the different versions did not have significantly different sensitivity or specificity levels (Table 4). Additional validity tests were performed on stratified samples based on sex, education, and age groups: these results did not indicate any marked differences in all measured values of validity within the different strata. However, it should be noted that the statistical power for these tests was limited.

**Table 3:** Validity tests of the three versions of the test on data from HARMONY and ADAMS

		Clinical diagnosis		Sensitivity	Specificity	PPV^a^	NPV^b^
		+	−	%	%	%	%
HARMONY							
Full MMSE	+	365	86	90.3	77.9	80.9	88.6
	−	39	304				
MMSE-SF	+	374	109	92.6	72.1	77.4	90.4
	−	30	281				
MMSE-SF-C	+	367	101	90.8	74.1	78.4	88.7
	−	37	289				
ADAMS							
Full MMSE	+	296	37	79.6	81.9	88.9	68.7
	−	76	167				
MMSE-SF	+	296	35	79.6	82.8	89.4	69.0
	−	76	169				
MMSE-SF-C	+	274	25	73.7	87.7	91.6	64.6
	−	98	179				

A cutoff <24 was applied to the full version and <8 to the two abridged tests

^a^Positive predictive value

^b^Negative predictive value

**Table 4:** Significance test for testing if the sensitivity levels are equal (H0: *p*1 = *p*2)

	Sensitivity	*p*	Specificity	*p*	PPV^a^	*p*	NPV^b^	*p*
HARMONY								
Full MMSE, MMSE-SF	90.3, 92.6	0.32	77.9, 72.1	0.07	80.9, 77.4	0.20	88.6, 90.4	0.52
Full MMSE, MMSE-SF-C	90.3, 90.8	0.90	77.9, 74.1	0.24	80.9, 78.4	0.41	88.6, 88.7	1.00
MMSE-SF, MMSE-SF-C	92.6, 90.8	0.44	72.1, 74.1	0.57	77.4, 78.4	0.75	90.4, 88.7	0.52
ADAMS								
Full MMSE, MMSE-SF	79.6, 79.6	1.00	81.9, 82.8	0.90	88.9, 89.4	0.90	68.7, 69.0	1.00
Full MMSE, MMSE-SF-C	79.6, 73.7	0.07	81.9, 87.7	0.13	88.9, 91.6	0.28	68.7, 64.6	0.35
MMSE-SF, MMSE-SF-C	79.6, 73.7	0.07	82.8, 87.7	0.21	89.4, 91.6	0.42	69.0, 64.6	0.31

^a^Positive predictive value

^b^Negative predictive value

The ROC curves for predicting dementia with the full version of the MMSE and the two abridged tests showed similar results for all three versions (Supplementary material). The three versions had the following unadjusted AUC values: full MMSE = 0.87, MMSE-SF = 0.89 and MMSE-SF-C = 0.89. After adjusting for gender, age, and education the AUC values were: full MMSE = 0.85, MMSE-SF = 0.87 and MMSE-SF-C = 0.86.

## Discussion

The aim of this study was to validate two abridged versions of the MMSE. The results show that both versions had validity comparable to the full MMSE in relation to the clinical diagnoses. These findings were consistent in both the Swedish (HARMONY) and the US (ADAMS) data.

A limitation of the study is that the two abridged tests were not collected independently from the participants in HARMONY and ADAMS, but instead the items were derived from the full original MMSE tests that were administered to the participants in those studies. Additional limitations stem from the restricted inclusion criteria in the ADAMS sample. Many participants were excluded due to missing items in the original MMSE and, more importantly, they were not missing at random, as a large share belonged to the dementia category.

However, a prominent feature of the abridged tests is that in some contexts, they can achieve a higher response rate (Fong et al. 2009). This involves both the actual participation in a test as well as the probability of completing all items. That is, the likelihood that a frail individual will participate in a test may be dependent on length and scope of the test. The complementary version (MMSE-SF-C) may therefore enable interviews with groups that are not available for face-to-face interviews and may also be appropriate for subjects with vision impairment or other physical impairments. While the two tests may have slightly different applications, they were both comparable and performed equally well. An additional benefit of these validated scales in comparison to other available short scales, e.g., TICS (Brandt et al. 1988) and COGTEL (Kliegel et al. 2007), is that they are comparable to those of the MMSE. Although the original MMSE has been shown to be imprecise in differentiating between clear-cut dementia cases and cases of questionable cognitive impairment (Mitchell 2013), it is still the most widely used test. This therefore allows for comparisons both between studies and nations. With the exception of the high sensitivity levels in HARMONY, the validity levels were moderate for the two abridged tests but comparable with the original MMSE. The lower sensitivity rates (for all versions of the test) in the ADAMS sample compared with the HARMONY sample can probably be attributed to the difference in proportions in the questionable dementia/CIND categories, reflecting differences in the criteria for questionable dementia and CIND. Adjustments of age, gender, and education lowered the validity of all tests somewhat, but there was no loss of predictive precision when using the abridged forms rather than the full MMSE. This ultimately means that short scales are comparable with the full-length version.

Even if the original MMSE is relatively quick to administer it might still be too demanding for older people taking part in an already lengthy study. Our findings suggest that these two abridged versions of the MMSE have adequate validity and perform well against the original MMSE, and may therefore be feasible alternatives that can be helpful in reaching more participants and to ensure that samples are more representative of the population. The abridged versions could therefore be alternatives worthy to consider in larger population studies where interview length is restricted and the respondent burden is high.

## Electronic supplementary material

Below is the link to the electronic supplementary material.

Supplementary material 1 (DOCX 89 kb)
