# Supplementary material for: Validation of abridged mini-mental state examination scales using population-based data from Sweden and USA
Source: Eur J Ageing. 2016 Aug 20;14(2):199–205. doi: 10.1007/s10433-016-0394-z (PMC5435785; doi:10.1007/s10433-016-0394-z)
Supplement: Supplementary file 1 — Supplementary material 1 (DOCX 89 kb) [file 10433_2016_394_MOESM1_ESM.docx]

0

1

TPR

0

1

FPR

Full MMSE

MMSE-SF

MMSE-SF-C

**HARMONY**

Figure 1 & 2. Displays the ROC curves for the three versions of the scales, the full MMSE, MMSE-SF and MMSE SF-C.

*Notes:* TPR = True Positive Rate/Sensitivity, FPR = False Positive Rate/Specificity.
